# Clinical and microbiological characteristics and challenges in diagnosing infected aneurysm: a retrospective observational study from a single center in Japan

**DOI:** 10.1186/s12879-022-07567-0

**Published:** 2022-06-30

**Authors:** Kohsuke Matsui, Kensuke Takahashi, Masato Tashiro, Takeshi Tanaka, Koichi Izumikawa, Takashi Miura, Kiyoyuki Eishi, Akitsugu Furumoto, Koya Ariyoshi

**Affiliations:** 1 Department of Infectious Diseases, Nagasaki University Hospital, 1-7-1 Sakamoto, Nagasaki-shi, Nagasaki 852-8501 Japan; 2 Infection Control and Education Center, Nagasaki University Hospital, 1-7-1 Sakamoto, Nagasaki-shi, Nagasaki 852-8501 Japan; 3 Department of Cardiovascular Surgery, Nagasaki University Hospital, 1-7-1 Sakamoto, Nagasaki-shi, Nagasaki 852-8501 Japan; 4 Infectious Diseases Experts Training Center, Nagasaki University Hospital, 1-7-1 Sakamoto, Nagasaki-shi, Nagasaki 852-8501 Japan

**Keywords:** Infected aneurysm, Aortic aneurysm, Misdiagnosis

## Abstract

**Background:**

It is challenging to diagnose infected aneurysm in the early phase. This study aimed to describe the clinical and microbiological characteristics of infected aneurysm, and to elucidate the difficulties in diagnosing the disease.

**Methods:**

Forty-one cases of infected aneurysm were diagnosed in Nagasaki University Hospital from 2005 to 2019. Information on clinical and microbiological characteristics, radiological findings, duration of onset, and type of initial computed tomography (CT) imaging conditions were collected. Factors related to diagnostic delay were analyzed by Fisher’s exact test for categorical variables or by the Wilcoxon rank-sum test for continuous variables.

**Results:**

Pathogens were identified in 34 of 41 cases; the pathogens were Gram-positive cocci in 16 cases, Gram-negative rods in 13 cases, and others in five cases. Clinical characteristics did not differ in accordance with the identified bacteria. At the time of admission, 16 patients were given different initial diagnoses, of which acute pyelonephritis (n = 5) was the most frequent. Compared with the 22 patients with an accurate initial diagnosis, the 19 initially misdiagnosed patients were more likely to have been examined by plain CT. The sensitivities of plain CT and contrast-enhanced CT were 38.1% and 80.0%, respectively.

**Conclusions:**

In cases of infected aneurysm, diagnostic delay is attributed to non-specific symptoms and the low sensitivity of plain CT. Clinical characteristics of infected aneurysm mimic various diseases. Contrast-enhanced CT should be considered if infected aneurysm is suspected.

## Background

Infected aneurysm is an infectious disease that involves the aortic or arterial walls. The diagnosis of infected aneurysm is based on systemic signs of inflammation and aneurysmal change in the aorta or artery, with signs of infected aneurysm such as absence of intimal calcification, perianeurysmal fluid, or gas in the aortic wall revealed by computed tomography (CT), magnetic resonance imaging, or ultrasonography [1]. However, it is not always straightforward to accurately diagnose infected aneurysm in the early phase. A previous study revealed that only one-third of infected aneurysm cases are diagnosed accurately in the emergency department [2]. Accurate diagnosis in the early phase of the disease is essential because infected aneurysm has a reported mortality of 9–36% [2–8], and this mortality is partly attributable to misdiagnosis [9].

One of the reasons for the difficulty in diagnosing infected aneurysm may be the wide variation in causative pathogens. The typical classical pathogens causing infected aneurysm are non-typhoidal *Salmonella* and *Staphylococcus* spp., which account for 28–41% and 15–25% of cases, respectively [10–12]. However, causative bacteria vary among studies. Tsai et al. reported that 92% of infected aneurysms are caused by Gram-negative rods (GNR), including *Escherichia coli*, *Klebsiella* spp., and *Salmonella* spp., while *Staphylococcus aureus* accounts for only 8% [2]. In contrast, Brossier et al. reported that *Campylobacter* spp. and *Streptococcus pneumoniae* are the two most common pathogens in infected aneurysms [13].

Another possible reason for the difficulty in diagnosing infected aneurysms is difficulties in radiological diagnosis. According to the Organisation for Economic Co-operation and Development data, the number of CT scanners in Japan is 111 per 1,000,000 inhabitants, which is the highest among countries that are members of the Organisation for Economic Co-operation and Development [14]. Due to the excellent accessibility to CT scanners and universal health insurance coverage in Japan, plain CT is often performed as an initial radiological investigation for patients with unknown sources of fever. However, to our knowledge, the sensitivity of plain CT has not been reported, although this would be of great interest to clinicians with good access to CT scanners. We hypothesized that some cases of infected aneurysm are overlooked by initial plain CT and misdiagnosed, leading to diagnostic delay.

The aims of this study were to describe the clinical and microbiological characteristics of infected aneurysm, and to elucidate the difficulties in diagnosing the disease.

## Methods

The study design was a single-center, retrospective case series based on the medical records of Nagasaki University Hospital, which is a tertiary referral hospital in Nagasaki, Japan. We searched the hospital medical records, administration records, and operation records of the Department of Cardiovascular Surgery, and included patients diagnosed with infected aneurysm from April 2005 to December 2019. In our study, infected aneurysm was defined by clinical evidence of acute infection (fever, elevated white blood cell count, or elevation of C-reactive protein concentration) and imaging findings compatible with infected aneurysm of either the aorta or an artery accompanied by one of the following findings: positive blood culture, positive aortic or arterial wall culture, operative findings compatible with infected aneurysm, pathological findings consistent with infected aneurysm, or improvement of the aneurysm by administration of antibiotics. Cases with infection of a prosthetic graft or stent, aorto-enteric fistula, or intracranial artery, and secondary infection due to trauma or operation were excluded.

Our diagnostic and therapeutic approach was as follows. Infected aneurysm was suspected when a patient had signs of acute inflammation with typical radiological findings of infected aneurysm such as rapid aneurysm development, saccular aneurysm, periaortic or periarterial fat density, or a soft tissue mass around the aorta or artery. For all patients with suspected infected aneurysm, blood cultures were taken and infectious disease physicians were consulted. Positive blood cultures were subcultured on blood agar, chocolate agar, bromothymol blue agar, and chromogenic agar. Columbia blood agar was used if an anaerobic blood culture bottle was positive. Bacterial identification was made using the BD Phoenix 100™ system (Beckton Dickinson, USA) before 2012, while the MALDI Biotyper^®^ system (Bruker Daltonics, Germany) was used from 2013 onwards. Drug susceptibility testing was done using the BD Phoenix 100™ system (Beckton Dickinson, USA). All cases with positive blood cultures were reported to infectious disease physicians. Antibiotics effective against both *Staphylococcus aureus* and *Enterobacteriaceae* spp. were chosen as an initial empirical treatment. If causative pathogens were identified, antibiotics were subsequently streamlined based on the results of drug susceptibility testing. In cases without detection of causative pathogens, definitive antibiotics were selected by infectious diseases consultants based on the severity and clinical course. Open surgery, endovascular intervention, or conservative treatment were chosen at the surgeons’ discretion. When open surgery was performed, the aortic or arterial wall was sent for both pathologic examination and culture.

For each patient, the following information was collected: age, sex, underlying diseases, initial diagnosis at admission, clinical features, laboratory findings, causative microorganism, radiological findings, clinical course until diagnosis, antibiotic and invasive treatment, and in-hospital outcome. Onset was defined as the date that infected aneurysm-related symptoms, including fever, localized pain, or anatomy-specific symptoms such as hemoptysis or hematuria, were recognized by either patients or clinicians. All patients were followed up during hospitalization in Nagasaki University Hospital. There was no loss to follow-up.

Qualitative factors related to the accuracy of the first imaging study were analyzed by Fisher’s exact test, while quantitative factors were analyzed by the Wilcoxon rank-sum test. Sensitivities of plain and contrast-enhanced CT in the diagnosis of infected aneurysm were obtained by calculating the proportions of patients who were diagnosed accurately by initial CT under each type of CT condition. Location of infection, causative pathogen, type of initial CT, days from onset to initial CT, days from onset to diagnosis, and in-hospital mortality were analyzed as factors potentially associated with the accuracy of diagnosis by first CT. Missing data were categorized as “unknown”. All analyses were performed with STATA, version 17.0 (STATA Corp). A *P* value of less than 0.05 was defined as statistically significant.

## Results

### General characteristics

**Fig. 1:**
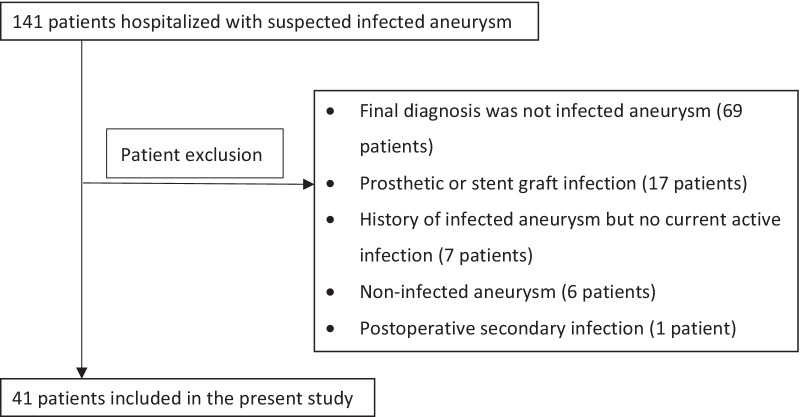
Flow diagram of patient selection

From April 2005 to December 2019, 141 patients were admitted to our hospital with suspicion of infective aneurysm. Among those patients, 41 fulfilled the case definition of infected aneurysm (Fig. 1). Patient characteristics are shown in Table 1. Among the 41 patients with infected aneurysm, 87.8% of patients had at least one factor related to atherosclerosis. One patient had aneurysms that simultaneously developed at the thoracic aorta and femoral artery. The predominant symptoms were fever and localized pain at the affected aorta or artery, although 26.8% of patients had no fever before admission. The median duration from onset to diagnosis was 14 days (range 0 to 76 days). In cases with causative pathogens detected, the most frequently used definitive antibiotic based on the results of drug susceptibility testing was penicillin, followed by cephalosporin, fluoroquinolone, combinations of anti-methicillin-resistant *S. aureus* (MRSA) and beta-lactam antibiotics, anti-MRSA antibiotics alone, and carbapenem. Open surgery and endovascular intervention were performed in 63.4% and 17.1% of cases, respectively. Three patients died during hospitalization, giving an in-hospital mortality rate of 7.3%. Among the three patients who died, one underwent open repair, and two were considered to have contraindications to further intervention.

**Table 1:** Clinical characteristics of patients with infected aneurysm

Characteristics	Value (n, %)	
	All cases (n = 41)	Cases caused by GNR other than *Salmonella* (n = 12)
Age in years, median (IQR)	71 (64–78)	72 (70.5–79)
Male sex	33/41 (80.5%)	8/12 (66.7%)
Underlying conditions		
Hemodialysis	7/41 (17.1%)	1/12 (8.3%)
Diabetes	11/41 (26.8%)	3/12 (25.0%)
Hypertension	25/41 (61.0%)	5/12 (41.7%)
Current cigarette smoker	13/41 (31.7%)	2/12 (16.7%)
CVD	18/41 (43.9%)	4/12 (33.3%)
Location of aneurysm*		
Thoracic aorta	12/42 (29.3%)	4/12 (33.3%)
Thoracoabdominal aorta	6/42 (14.6%)	1/12 (8.3%)
Abdominal aorta	15/42 (36.6%)	5/12 (41.7%)
Iliac artery	5/42 (12.2%)	1/12 (8.3%)
Femoral artery	2/42 (4.9%)	0/12 (0%)
Ulnar artery	1/42 (2.4%)	0/12 (0%)
Celiac artery	1/42 (2.4%)	1/12 (8.3%)
Symptoms		
Fever	33/41 (80.5%)	10/12 (83.3%)
Pain at affected site	29/41 (70.1%)	4/12 (33.3%)
Abdomen/flank	11/41 (26.8%)	1/12 (8.3%)
Back/lower back	12/41 (29.3%)	1/12 (8.3%)
Leg/inguinal region	6/41 (14.6%)	2/12 (16.7%)
Hemoptysis	4/41 (9.8%)	2/12 (16.7%)
Cough	2/41 (4.9%)	1/12 (8.3%)
Tarry stools	1/41 (2.4%)	1/12 (8.3%)
Shock	2/41 (4.9%)	0/12 (0%)
Respiratory failure	9/41 (22.0%)	4/12 (33.3%)
Impaired consciousness	5/41 (12.2%)	2/12 (16.7%)
Days from onset to diagnosis, median (IQR)	14 (5–28)	16 (7–25)
Diagnosed by initial CT scan, median (IQR)	22/41 (53.7%)	6/12 (50.0%)
Intervention		
Open surgery	26/41 (63.4%)	8/12 (66.7%)
Endovascular procedure	7/41 (17.1%)	2/12 (16.7%)
None	8/41 (19.5%)	2/12 (16.7%)
Days of hospitalization, median (IQR)	36 (24.8–50)	38 (31–54)
In-hospital mortality	3/41 (7.3%)	1 (8.3%)

The table shows the clinical characteristics, treatment, and outcome of all patients with infected aneurysm, and of patients with infected aneurysm caused by Gram-negative rods (GNR) other than *Salmonella* spp.

*IQR* interquartile range, *CVD* cardiovascular diseases including stroke and ischemic heart diseases, *CT* computed tomography

*One patient with infected aneurysms in both the thoracic aorta and femoral artery was counted twice

### Causative pathogens

Microbiological, pathological, or serological signs of specific microorganisms were revealed in 33 cases (Table 2). These signs included a positive blood culture in 25 cases, pathological signs of infection in four cases, positive tissue culture of the aortic wall in three cases, and positive polymerase chain reaction of the aortic wall in one case. Among 34 causative pathogens, 16 were Gram-positive cocci (GPC). Seven cases were caused by *S. aureus*, including six cases of MRSA, the most frequent among all pathogens. Among 13 identified GNR, three were *E. coli* and three were *K. pneumoniae*, including one extended-spectrum beta-lactamase-producing *E. coli*. One case had co-infection of *K. pneumoniae* and MRSA. Only one case was caused by *Salmonella* spp. Other categories of microorganisms included *Treponema pallidum*, *Mycobacterium tuberculosis*, *Listeria monocytogenes*, and undetermined Gram-positive bacteria. There were no cases of fungal infection.

**Table 2:** Causative bacteria classified by Gram stain findings

Gram stain findings	Pathogens	n = 42*
GPC (n = 16)	*Staphylococcus aureus*	7
	*Streptococcus pneumoniae*	2
	*Streptococcus pyogenes*	2
	*Streptococcus dysgalactiae*	1
	*Streptococcus mitis*	1
	*Streptococcus sanguis*	1
	*Streptococcus acidominimus*	1
	Undetermined *Streptococcus* spp.	1
GNR (n = 13)	*Escherichia coli*	3
	*Klebsiella pneumoniae*	3
	*Enterobacter cloacae*	1
	*Citrobacter koseri*	1
	*Aeromonas hydrophilia*	1
	*Edwardsiella tarda*	1
	*Salmonella choleraesuis*	1
	Undetermined GNR	2
Others (n = 5)	*Treponema pallidum*	1
	*Mycobacterium tuberculosis*	1
	*Listeria monocytogenes*	1
	Undetermined Gram-positive bacteria	2
Undetected (n = 8)		

*GPC* Gram-positive cocci,* GNR* Gram-negative rods

*One case caused by two pathogens was counted twice

### Diagnoses at admission

Among the 38 community-acquired cases, only 16 (42.1%) patients were diagnosed accurately at admission (Table 3). Among the 22 patients whose initial diagnosis was not infected aneurysm, 16 patients were given a variety of different diagnoses such as acute pyelonephritis or infected endocarditis, while six patients were recognized as having disease with unknown causes.

**Table 3:** Diagnosis at admission of 38 patients with community-acquired infected aneurysms

Diagnosis at admission	Total (n = 38)
Infected aneurysm	16/38 (42.1%)
Others	22/38 (57.9%)
Acute pyelonephritis	5
Infective endocarditis	2
Aortic dissection	1
Aortic aneurysm without infection	1
Acute cholangitis	1
Bacteremia	1
Vertebral osteomyelitis	1
Retroperitoneal abscess	1
Pneumonia	1
Lung tumor	1
Gastric ulcer	1
No confirmed diagnosis at admission	6

### Factors associated with misdiagnosis

In all cases, CT was performed as a screening imaging test to investigate the causes of fever or pain. Diagnosis of infected aneurysm was correctly made based on initial CT in 22 cases, but was misdiagnosed in 19 cases. Factors associated with misdiagnosis at the time of the initial CT scan are summarized in Table 4. Data for the type of initial CT were not available in five cases.

**Table 4:** Univariate analysis of factors and outcomes related to the diagnostic accuracy of initial CT

	Not diagnosed by initial CT (n = 19)	Diagnosed by initial CT (n = 22)	p
Location of infection*			0.962
Thoracic aorta	6/19 (31.6%)	6/23 (26.1%)	
Thoracoabdominal aorta	3/19 (15.8%)	3/23 (13.0%)	
Abdominal aorta	7/19 (36.8%)	8/23 (34.8%)	
Iliac artery	2/19 (10.5%)	3/23 (13.0%)	
Others	1/19 (5.3%)	3/23 (13.0%)	
Pathogen**			0.577
GPC	9/20 (45.0%)	9/22 (40.9%)	
GNR	7/20 (35.0%)	6/22 (27.3%)	
Others	2/20 (10.0%)	1/22 (4.5%)	
Undetected	2/20 (10.0%)	6/22 (27.3%)	
Type of initial CT			0.042
Plain	13/19 (68.4%)	8/22 (36.4%)	
Contrast-enhanced	3/19 (15.8%)	12/22 (54.5%)	
Unknown	3/19 (15.8%)	2/22 (9.1%)	
Days from onset to initial CT, median (IQR)	6 (2–9)	7.5 (2.3–22.3)	0.254
Days from onset to diagnosis, median (IQR)	21 (9–32.5)	7.5 (2.3–22.3)	0.051
In-hospital mortality	1/19 (5.3%)	2/22 (9.1%)	

*CT* computed tomography, *GPC* Gram-positive coccus, *GNR* Gram-negative rod, IQR: interquartile range

*One patient with infected aneurysms in both the thoracic aorta and femoral artery was counted twice

**One patient with infected aneurysm caused by two pathogens was counted twice

Patients who underwent contrast-enhanced CT were more likely to be diagnosed accurately with infected aneurysm. The sensitivities of plain and contrast-enhanced CT were 38.1% and 80.0%, respectively. The diagnostic accuracy of the initial CT examination was not influenced by the causative pathogen or location of the affected aorta or artery. While the median duration from onset to initial CT was similar, the duration from onset to accurate diagnosis tended to be longer in patients who were not diagnosed by initial CT than in those who were diagnosed by initial CT, although the difference was not statistically significant (Table 4). There were three reasons for the inaccurate diagnosis by initial CT. Aneurysm itself was not noted in six patients on plain CT and in two patients on contrast-enhanced CT. Inflammation sign was not detected in four patients on plain CT and one patient on contrast-enhanced CT, although aneurysms were noted in these patients. In three patients assessed with plain CT, abnormal findings were identified, but were considered to be diseases other than infected aneurysm.

## Discussion

**Fig. 2:**
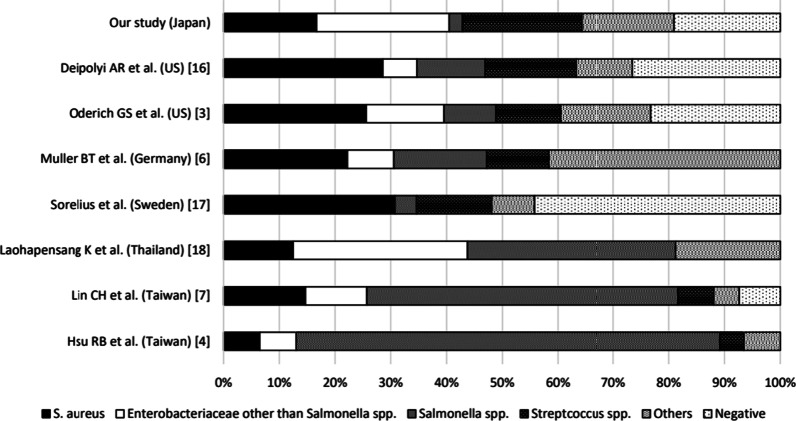
Comparison of causative bacteria in previous studies and our study. Proportion of cases of infected aneurysm caused by *Staphylococcus aureus*, *Enterobacteriaceae* other than *Salmonella* spp., *Salmonella* spp., *Streptococcus* spp., other bacteria, and cases without detection of causative bacteria in previous studies and our study

Our study described the clinical and bacteriological characteristics of infected aneurysm among patients admitted to our institute in the past 15 years. To our knowledge, this is the largest case series of infected aneurysm in Japan. In our study, the numbers of infected aneurysm cases caused by GPC and GNR were almost equal. Among the GNR cases, only one was caused by *Salmonella* spp., while more than half were due to either *E. coli* or *K. pneumoniae*. The reason that there was only one case due to *Salmonella* might be partially due to a decrease in the number of *Salmonella* infections in Japan, as the number of enteritis cases due to food poisoning caused by *Salmonella* spp. showed a dramatic decrease from 16,576 to 1996 to 861 in 2020 [15]. In contrast, a previous case series in Japan that included patients treated from 1982 to 2009 showed that two of 11 cases (18%) of infected aneurysm were caused by *Salmonella* spp. Although the number of cases is too small to draw a definitive conclusion, a comparison between the proportion of *Salmonella* cases in the previous case series and our study supports our hypothesis that the very small number of patients with infected aneurysm due to *Salmonella* spp. is due to the decrease in the number of *Salmonella* infections in Japan. With regard to GNR, our study showed a relatively higher proportion of *Enterobacteriaceae* other than *Salmonella* spp. than that reported in studies in European countries and North America, and a similar proportion to a study in Thailand (Fig. 2) [3, 4, 6, 7, 16–18]. The prevalence of GNR bacteremia reportedly shows a negative correlation with latitude [19]. Therefore, one of the reasons for the different proportion of *Enterobacteriaceae* other than *Salmonella* spp. in our study compared with previous studies is likely to be a geographical factor.

Previous reports have suggested relationships between specific pathogens and outcomes. The rupture and mortality rates of infected aortic aneurysm caused by GNR are reportedly higher than the rates for infected aneurysm caused by GPC [10]. However, another study showed that having *Salmonella* spp. as the causative pathogen is a negative predictor of mortality in patients with infected aortic aneurysm [4]. In our study, the clinical characteristics of all cases and cases caused by GNR other than *Salmonella* spp. were similar. Among our three mortality cases, the cause of the infected aneurysm was GPC in two cases and undetermined GNR in one case. The small number of mortality cases in our study makes it difficult to analyze the association between pathogens and mortality.

More than half of our patients were not initially diagnosed with infected aneurysm. There may be several reasons for the inaccuracy of diagnosis at admission. First, the symptoms of infected aneurysm are not specific and can mimic other diseases. In our study, five patients were initially diagnosed with acute pyelonephritis, and one was diagnosed with acute cholangitis. The typical clinical symptoms of acute pyelonephritis are fever and low back pain, which are similar to those of infected aneurysm. There are some case reports of infected aneurysm initially diagnosed as acute pyelonephritis [20, 21]. It is possible that infected aneurysm occurs secondarily from acute pyelonephritis or acute cholangitis, but in four of our five patients initially diagnosed with pyelonephritis, those diagnoses were not accurate because the urine culture results were negative or inconsistent with the blood culture results. Clinicians should be aware of the non-specificity of the clinical manifestations of infected aneurysm, which can mimic other infectious diseases. Infected aneurysm should be one of the differential diagnoses when pyelonephritis or other infectious diseases show unfavorable clinical courses.

Second, although some aneurysms develop secondarily from other infected sources through the bloodstream, clinicians tend to overlook the secondary infection while treating primary infection. Two of our patients who had definite diagnoses of infective endocarditis developed subsequent infected aneurysm of the femoral and ulnar artery, respectively. Those aneurysms became apparent during the treatment of infective endocarditis. Even if the initial diagnoses are correct, careful observation for possible secondary infected aneurysm is needed, especially in patients with bloodstream infections such as infective endocarditis.

Third, the interpretation of imaging studies for infected aneurysm is not straightforward. Our study revealed that screening for the source of infections using plain CT overlooks many cases of infected aneurysm. Several studies have evaluated radiologic modalities for detecting infected aneurysm. A study reported in 2020 showed that the sensitivities of contrast-enhanced CT and positron emission tomography-CT for infected aortic aneurysm are 63–88% and 85–100%, respectively [22]. A meta-analysis that included infection of either the aorta or cerebral arteries showed that the sensitivities of contrast-enhanced CT and magnetic resonance imaging are 82% and 79%, respectively [23]. The sensitivity of contrast-enhanced CT in detecting infected aneurysm in our study was similar to that reported in previous studies. Although contrast-enhanced CT is the ideal choice to diagnose infected aneurysm, many clinicians prefer plain CT over contrast-enhanced CT for the initial investigation of the causes of fever or pain due to the potential risks of contrast-induced nephropathy and allergic reaction.

When we retrospectively reassessed the initial CT images of nine patients whose initial CT failed to show the infected aneurysm, subtle signs of inflammation were recognized in six cases. The early sign of infected aortic aneurysm is an increase in fat density around the aorta [24]. When plain CT is performed, clinicians should thoroughly check abnormal findings around the aorta and maintain appropriate communication with radiologists regarding the possibility of infected aneurysm in patients with fever of unknown causes. In addition, contrast-enhanced CT is crucial when there is a possibility of infected aneurysm.

It is notable that the in-hospital mortality rate in our study was 7.3%, which was lower than previous reports. The reported risk factors for death among patients with infected aneurysm include advanced age, non-*Salmonella* infection, no intervention, extensive peri-aortic infection, female sex, *Staphylococcus* infection, suprarenal aneurysm, treatment for spinal disease before being diagnosed, and connective tissue diseases [3, 4, 9]. However, the low mortality rate of our study does not appear to be attributable to these risk factors. One possible contributing factor is the difference in the time of diagnosis and treatment of patients. We included patients who were diagnosed with infected aneurysm from 2005 to 2019. In contrast, studies with an in-hospital mortality of higher than 20% included patients who were diagnosed in the 1990s [4, 6, 7, 9, 13, 25]. The mortality of both aortic aneurysm and sepsis have decreased over time, partly due to progressions in the treatment of both conditions [26, 27]. We believe that the lower mortality in our study than in previous studies was because of the improved management of infected aneurysm.

There are some limitations to our study. First, due to the retrospective nature of the study, some clinical information was unavailable. For example, not all initial CT images were accessible, especially when the initial CT was performed in other hospitals. Second, as most patients were transferred to other non-acute care hospitals after stabilization, the exact duration of treatment and long-term outcome were not available.

Third, the microbiological characteristics in our study are not necessarily generalizable to other settings, as these characteristics were probably affected by factors such as geographical factors, hospital level, and endemic bacteria. However, the difficulties in the diagnosis of infected aneurysm are generalizable because of the non-specific clinical features of this disease.

## Conclusion

Our study showed that a significant proportion of infected aneurysms were caused by GNR other than *Salmonella* spp., although the clinical characteristics in these cases were similar to those in cases caused by other bacteria. The diagnosis of infected aneurysm is challenging due to its non-specific clinical manifestations and wide spectrum of radiological findings. Contrast-enhanced CT plays a crucial role in the diagnosis of infected aneurysm.

## Data Availability

The datasets used and analyzed in the current study are available from the corresponding author on reasonable request.

## Abbreviations

CT: Computed tomography
GPC: Gram-positive coccus
GNR: Gram-negative rod
MRSA: Methicillin-resistant *Staphylococcus aureus*
